# Dislocation of Vertebræ

**Published:** 1874-01

**Authors:** 


Dislocation of Vertebræ.—Dr. J. W. Brooks, of Chicago, reports the following interesting case in the November number:

September 26th, 1872, I was requested hurriedly to visit F. B., a delicate, pale boy of eight or nine years. On arriving I, learned that on the previous evening, while playing at somersault in his father's parlor, he had injured his neck. Examination revealed a sub-atlo-axoid luxation. The chin was directed a little upward and to the right; the head was immobile; there was some numbness below the seat of injury, in all the members of the body; he was exceedingly nervous; there was no intense pain, but, as he expressed it, "it hurt all the time;" with a pale, damp, cool skin. With an assistant to steady and hold down the shoulders, and standing directly behind him (he being half recumbent), with a hand on either side of the cranium, and the fore-finger separated from the middle finger so that one fore-finger of each hand would come before the articulation, and the middle finger of each hand behind, I proceeded to rotate carefully, and at the same time to make counter-extension; presently an audible click announced the return of the parts to their normal position. Convalescence was soon established.

